# Functional Analysis of the VirSR Phosphorelay from *Clostridium perfringens*

**DOI:** 10.1371/journal.pone.0005849

**Published:** 2009-06-09

**Authors:** Jackie K. Cheung, Milena M. Awad, Sheena McGowan, Julian I. Rood

**Affiliations:** Department of Microbiology, Monash University, Clayton, Victoria, Australia; Max Planck Institute for Infection Biology, Germany

## Abstract

Toxin production in *Clostridium perfringens* is controlled by the VirSR two-component signal transduction system, which comprises the VirS sensor histidine kinase and the VirR response regulator. Other studies have concentrated on the elucidation of the genes controlled by this network; there is little information regarding the phosphorelay cascade that is the hallmark of such regulatory systems. In this study, we have examined each step in this cascade, beginning with autophosphorylation of VirS, followed by phosphotransfer from VirS to VirR. We also have studied the effects of gene dosage and phosphorylation *in vivo*. We have used random and site-directed mutagenesis to identify residues in VirS that are important for its function and have identified a region in the putative sensory domain of VirS that appeared to be essential for function. *In vitro* phosphorylation studies showed that VirSc, a truncated VirS protein that lacked the *N*-terminal sensory domain, was capable of autophosphorylation and could subsequently act as a phosphodonor for its cognate response regulator, VirR. Conserved residues of both VirS and VirR, including the D57 residue of VirR, were shown to be essential for this process. By use of Targetron technology, we were able to introduce a single copy of *virR* or *virR_D57N_* onto the chromosome of a *virR* mutant of *C. perfringens*. The results showed that *in vivo*, when *virR* was present in single copy, the production of wild-type levels of perfringolysin O was dependent on the presence of *virS* and an unaltered D57 residue in VirR. These results provide good evidence that phosphorylation is critical for VirR function.

## Introduction

*Clostridium perfringens* is a Gram-positive, endospore-forming, anaerobic pathogen that is the primary causative agent of gas gangrene or clostridial myonecrosis, and other human and animal diseases [1], [2]. The production of the extracellular toxins, α-toxin and perfringolysin O, which act synergistically in the gas gangrene disease process [3], [4], [5], [6], has been shown to be regulated by the VirS sensor histidine kinase and its cognate response regulator, VirR [1], [7], [8], [9]. VirR is a transcription activator that directly regulates the production of perfringolysin O, the cysteine protease α-clostripain, and the regulatory RNA molecules, VR-RNA, VirT and VirU [9], [10], [11], [12], [13]. It is through VR-RNA that the VirSR system indirectly regulates the production of α-toxin, collagenase (κ-toxin) [9], β2-toxin [14], a putative collagen adhesin [14], a cell-wall anchored DNase [15], and several housekeeping genes [9], [10], [16], [17].

The VirS sensor histidine kinase is predicted to contain six or seven transmembrane domains in its *N*-terminal region. Its *C*-terminal region contains several conserved motifs typical of histidine kinases, including the proposed site of autophosphorylation, H255, and the G box, which is thought to be involved in ATP binding [7], [18]. It is postulated that upon detection of an as yet unidentified signal by the *N*-terminal sensor region, VirS autophosphorylates at H255 [7]. The phosphoryl group is then transferred to a conserved aspartate residue, D57, that is located in the *N*-terminal region of VirR. This region also contains the E8, D9 and K105 residues, which in other response regulators are highly conserved and form a phosphoacceptor pocket [19]. After phosphorylation, activated VirR regulates the expression of its target genes by binding to specific DNA binding sites [20] via its *C*-terminal DNA binding domain [21]. We previously showed that VirR activates transcription of the perfringolysin O structural gene, *pfoA*, by binding independently to two imperfect 12-bp directly repeated sequences, called VirR boxes, located upstream of the *pfoA* promoter [20]. The maintenance of the integrity and spatial organisation of these VirR boxes is crucial for optimal perfringolysin O production [22]. In the three sequenced *C. perfringens* genomes [12], [23], VirR boxes have been identified upstream of several other genes, including genes encoding VR-RNA and α-clostripain. We have shown that these VirR boxes are functional and that VirR recognises and binds to each of these alternative binding sites [12], [22].

Although the role of the VirSR system in toxin production has been established, the *N*-terminal region(s) required for VirS function have not been determined and the role of phosphorylation in transcriptional activation has not been demonstrated. In this paper we identify a region located within one of the putative transmembrane domains that appears to be required for VirS function and show, using *in vitro* phosphorylation studies, that a truncated form of VirS is able to undergo autophosphorylation and subsequently act as the phosphodonor for VirR. Using perfringolysin O production as a reporter system for VirR function, we showed that activation of transcription was dependent on the presence of VirS and the D57 residue of VirR. These studies demonstrated that phosphotransfer from VirS to VirR and gene dosage were important factors in the regulation of perfringolysin O production by VirR.

## Materials and Methods

### Bacterial strains, plasmids and growth conditions

Bacterial strains are listed in Table 1, while plasmids are listed in Table S1. *Escherichia coli* strains were grown at 37°C in 2×YT or SOC media [24] supplemented with either 100 µg ml^−1^ ampicillin or 30 µg ml^−1^ chloramphenicol. *C. perfringens* strains were grown at 37°C in fluid thioglycollate medium (FTG) (Difco), trypticase-peptone-glucose (TPG) broth [25], Brain Heart Infusion (BHI) broth (Oxoid) or nutrient agar (NA) [26]. Where indicated, NA supplemented with 50 µg ml^−1^ erythromycin (NAEm_50_) or 30 µg ml^−1^ chloramphenicol (NACm_30_) was used for selection purposes. To screen for phospholipase C production, *C. perfringens* transformants were grown on egg yolk agar [27] supplemented with 30 µg ml^−1^ chloramphenicol (EYACm_30_), whilst perfringolysin O activity was visualised on horse blood agar (HBA) [7]. All *C. perfringens* agar cultures were incubated under anaerobic conditions in 10% (v/v) H_2_, 10% (v/v) CO_2_ in N_2_.

**Table 1:** Bacterial Strains and Plasmids.

Strain	Characteristics	Reference
**Strains**		
***E. coli***		
DH5α	F-φ80Δ *lacZ*dM15Δ(*lacZYA*-*argF*)U169 *endA1recA1 hsdr17*(τ_κ_^−^m_κ_^+^)*deoR thi-1 supE44 gyrA96 relA1*	Bethesda Research Laboratories
BL21(DE3) (C43)	F'*ompT hsdS*_B_(r_B_“m_B_”) *gal dcm* (DE3) (C43)	[35]
Epicurian Coli® XL1-Red	*endA1 gyrA96 thi-1 hsdR17 supE44 relA1 lac mutD5 mutS mutT Tn10(Tet ^r^ )*	Stratagene
***C. perfringens***		
TS133	Strain 13 *virR* :: *Tc^R^*	[8]
JIR325	Strain 13 Nal^R^ Rif^R^	[7]
JIR4000	JIR4025 *virS*::Tn*916*	[7]
JIR12100	TS133 *plc* :: Targetron	This study
JIR12106	TS133 *plc* :: TargetronΩ*virR_D57N_*	This study
JIR12136	TS133 *plc* :: TargetronΩ*virR*	This study

### Perfringolysin O assay

The level of perfringolysin O activity in the culture supernatants was determined by a doubling dilution hemolysin assay using horse red blood cells, as previously described [3]. The titre was defined as the reciprocal of the last well that showed complete hemolysis.

### Molecular techniques

Plasmid DNA was isolated from *E. coli* [28] and *C. perfringens* [29] cells as previously described. DNA for sequencing was prepared as per the PRISM Big Dye Terminator Cycle Sequencing Ready Reaction Kit (Applied Biosystems). Restriction endonucleases and other enzymes were used as specified by the manufacturer (Roche Diagnostics, New England Biolabs). All oligonucleotide primers are listed in Table S2

Competent *E. coli* [30] and *C. perfringens* [31] cells were prepared and transformed as described previously, unless otherwise indicated. *C. perfringens* genomic DNA was isolated from 5 ml FTG broth cultures as before [32]. PCR amplification was carried out as before [22]. PCR products were purified either directly using QIAquick® PCR Purification Kit (Qiagen) or were extracted from agarose gels using the QIAquick® Gel Extraction Kit (Qiagen), according to the manufacturer's instructions.

### Random Mutagenesis

Random *virS* mutants were isolated after passage through the DNA repair deficient strain Epicurean Coli® XL1-Red (Stratagene). The target plasmid was pJIR884 [7], which contains an intact copy of the *virS* gene and the upstream *virR* promoter. Four independently derived pJIR884 DNA samples were obtained from transformed XL1-Red cells and used to transform *C. perfringens* strain JIR4000, a JIR325 derivative in which the *virS* gene has been insertionally inactivated by Tn*916* [7]. To isolate random mutations by chemical mutagenesis, pJIR884 was incubated in 1 M hydroxylamine in 1 mM EDTA (pH 6) for 30 to 180 min at 70°C. Plasmid DNA was then used to transform strain JIR4000. Irrespective of the mutagenesis method, *virS* mutants were detected as non-hemolytic colonies on HBA supplemented with 50 µg ml^−1^ erythromycin. Since perfringolysin O production is dependent upon the VirSR system [22], [33], an inability to complement the *virS* mutation in JIR4000, i.e. no hemolysis on HBA, was used as a direct means of detecting loss of VirS function. To facilitate analysis, plasmid DNA was recovered from all non-hemolytic strains of *C. perfringens* and used to transform *E. coli*. Sequence analysis was carried out on DNA isolated from the resultant *E. coli* strains to identify plasmids with mutations within the *virS* gene. Sequence analysis was used to identify plasmids with mutations within the *virS* gene. Selected plasmids derived from both methods of random mutagenesis were further analysed and are listed in Table 2.

**Table 2:** The effect of *virS* mutations on perfringolysin O production.

Strain	Base Change^b^/Characteristic	Amino Acid Change	PFO titre (log)_2_ a
**Random Mutants**			
JIR4000(pJIR751)	vector control		<1
JIR4000(pJIR884)	*virS^+^* positive control		5.8±0.1
JIR4000(pJIR2108)*	CTT → **T**TT	L99F	<1
JIR4000(pJIR1983)	ACA → **G**CA	T100A	<1
JIR4000(pJIR2105)*	GAA → **A**AA	E102K	<1
JIR4000(pJIR1977)	CAT → **T**AT	H255Y	<1
JIR4000(pJIR1930)	GAT → G**G**T	D256G	<1
JIR4000(pJIR2028)	AAT → AA**A**	N259K	<1
JIR4000(pJIR2106)*	CAC → **T**AC	H260Y	<1
JIR4000(pJIR2073)	TGT → T**A**T	C335Y	<1
JIR4000(pJIR1953)	GGC → G**A**C	G402D	<1
JIR4000(pJIR2186)*	GGA → **A**GA	G415R	<1
**Site-Directed Mutants**			
JIR4000(pJIR2306)	ATT → **T**TT	I88F	4.6±0.1
JIR4000(pJIR2305)	TTA → TT**T**	L92F	3.7±0.1
JIR4000(pJIR2326)	TTA → **G**CA	L96A	5.7±0.1
JIR4000(pJIR2325)	TTA → **AAT**	L96N	5.7±0.1
JIR4000(pJIR2206)	TTA → TT**T**	L96F	<1
JIR4000(pJIR2379)	CTT → **AA**T	L99N	<1
JIR4000(pJIR2378)	CTT → **GCA**	L99A	<1
JIR4000(pJIR2175)	ACA → **T**CA	T100S	5.0±0.5
JIR4000(pJIR2173)	ACA → **TGT**	T100C	2.0±0.4
JIR4000(pJIR2177)	ACA → **GT**A	T100V	<1
JIR4000(pJIR2231)	CTA → **T**T**T**	L104F	4.6±0.3

a PFO titre±SD: Perfringolysin O activity is shown as the average of duplicate assays carried out on preparations from at least three separate cultures of each strain.

b Base changes are shown in bold.

* refers to hydroxylamine derived mutants.

### Site-directed Mutagenesis

Site-directed mutagenesis of the *virS* gene carried on pJIR2056 was performed using a modification of the unique site elimination method [34] with the U.S.E. Mutagenesis Kit™ (Amersham Pharmacia Biotech), the GeneEditor™ *in vitro* Site-Directed Mutagenesis Kit (Promega) [21], or the PCR-based Quikchange™ XL Site-Directed Mutagenesis Kit (Stratagene). The *virS* genes and upstream promoter regions of the mutated plasmids were sequenced to confirm that only the desired mutation was present. To test the effect of the mutations on VirS function, a 3.8-kb *Xba*I/*Sal*I fragment from each mutated plasmid was isolated, cloned into the *Xba*I/*Sal*I site of the *E. coli/C. perfringens* shuttle vector pJIR751, and used to transform *C. perfringens* strain JIR4000. Site-directed mutagenesis of the *virR* gene and the cloning of the resultant mutated genes were carried out as described previously [21].

### Construction of expression plasmids

To overexpress VirSc and its derivatives, VirSc_H255I_ and VirSc_G402D_, a 0.69 kb PCR product was generated using the primers JRP1873 and JRP1133 (Table S2). These primers incorporated *Nde*I and *Xho*I sites at the 5′ and 3′ ends, respectively, which facilitated the insertion of the DNA fragment into the expression vector pET-22b(+) (Novagen) to construct pJIR2699, pJIR2792 and pJIR2825, respectively (Table S1). To overexpress *N*-terminal 6×His tagged VirR_D57N_, a 0.9 kb *Xba*I/*Hin*dIII fragment from pJIR1732 (Table S1) was subcloned into pRSETA (Invitrogen) to produce pJIR1747.

### Overexpression and purification of His-tagged proteins

To induce the expression of recombinant proteins, *E. coli* C43 (DE3) [35] derivatives were induced with 1 mM IPTG for 3 h at 37°C (VirSc,VirSc_H255I_ & VirSc_G402D_) or 1 h at 37°C (VirR & VirR_D57N_). Proteins were purified using Talon resin (Clonetech) and dialysed as described previously [33]. Protein concentration was determined with the bicinchoninic acid protein assay kit (Pierce).

### Autophosphorylation of VirSc and Phosphotransfer from VirSc to VirR

VirSc was diluted in phosphorylation buffer (50 mM HEPES [pH 8.0], 50 mM KCl, 5 mM MgCl_2_, 0.5 mM EDTA, 2 mM DTT) to a final concentration of 20 µM, and aliquoted for use in individual phosphorylation reactions. Phosphorylation was initiated by the addition of 0.1 volumes of a 10× reaction mixture containing either 2.5 µM [γ-^32^P]ATP (111TBq/millimole) (Perkin Elmer Life Sciences) and 247.5 µM unlabelled ATP, or 247.5 µM unlabelled ATP alone. The latter reaction mixture was added to unlabelled reactions that were subject to Western blot analysis using a Penta-His antibody (Qiagen). To examine autophosphorylation specificity, reactions were pre-incubated with 50 mM EDTA or 2.5 mM unlabelled ATP at room temperature (approx 23°C) for 5 min before the addition of the 10× reaction mixture. All reactions were incubated at room temperature for 5 min, stopped by the addition of an equal volume of 2× gel loading buffer, and then subjected to SDS-PAGE [36]. The labelled gel was then vacuum dried and exposed to X-ray film (Fuji).

For phosphotransfer studies, VirSc (10 µM) was autophosphorylated as above, in a total volume of 10 µl, with the exception that reactions were carried out in phosphotransfer buffer (50 mM MOPS [pH 7.0], 50 mM KCl, 10 mM MgCl_2_, 0.5 mM EDTA, 2 mM DTT) and incubated at room temperature for 1 h. VirR or VirR_D57N_ was then added to a final concentration of 0.9 µM, and reactions were incubated for a further 15 min at room temperature before the addition of 10× reaction mixture and incubation at room temperature for varying amounts of time. Proteins were separated by SDS-PAGE and gels were treated as before. The presence of proteins in these phosphorylation studies was verified by Western blot analysis using His-tag antibodies on duplicate reaction samples (data not shown).

### Isolation of *C. perfringens* cell extracts

Cell pellets derived from 1 ml TPG broth cultures were resuspended in 350 µl of 1× PBS, approximately 100 µl of 150–212 micron glass beads (Sigma-Aldrich) was added and the cells were lysed by vigorous agitation, twice for 45 sec, in a FastPrep FP120 Cell Disrupter (Savant/Bio101). Cell debris was removed by centrifugation at 12,000 g for 10 min at 4°C, and the soluble whole cell extracts were collected. Following determination of the protein concentration, 20 µg of each extract was used in Western blot analysis with VirR-specific antisera.

### Western blot analysis

Following SDS-PAGE, proteins were transferred to Protran nitrocellulose membrane (Schleicher and Schuell) using a mini Trans-Blot® Electrophoretic Transfer Cell (BioRad) for Western blot analysis. The nitrocellulose membrane was developed using Penta-His antibody (1∶2000 dilution) or VirR-specific antiserum (1∶1000 dilution) [21] and the Western Lightning Chemiluminescence Reagent (Perkin Elmer Life Sciences), in accordance with the manufacturer's instructions.

### Introduction of *virR* and *virR_D57N_* onto the chromosome by use of a Targetron

To construct the plasmids used to introduce *virR* and *virR_D57N_* onto the chromosome, the genes were PCR amplified using primers JRP2812 and JRP2813 (Table S2). The oligonucleotides introduced *Mlu*I sites that facilitated cloning into the unique *Mlu*I site within the *plc* target region of pJIR750ai [37], such that the genes were transcribed in the same direction as the intron. The resultant plasmids, pJIR3326 (*virR*) and pJIR3243 (*virR_D57N_*) were introduced into *C. perfringens* strain TS133 (Table 1) by electroporation as before [38]. Transformants were selected on NACm_30_ and several transformants were subcultured into FTG broth. After overnight incubation at 37°C, 1 ml of each culture was used to inoculate 20 ml of BHI broth and grown at 37°C for 4 h. Cultures were then serially diluted and subcultured onto EYACm_30_. After overnight incubation at 37°C, colonies that were not surrounded by a white zone of opalescence were selected for further analysis. These colonies represented cells in which the α-toxin gene, *plc*, had been disrupted by the Targetron. The insertion of the element was confirmed by PCR, using primers JRP2873 and JRP2874 (Table S2). To cure the strains of the replicating plasmid constructs, the strains were passaged twice a day in 20 ml of BHI broth for five days. Single colonies were isolated and those that were chloramphenicol sensitive were selected for further analysis. Southern blots were carried out as previously described [37] using DIG-labelled *catP*, *virR* and *plc* probes to confirm the insertion of the genes on the chromosome and the loss of the Targetron plasmid constructs (data not shown).

### Isolation of RNA and Quantitative Real Time (QRT)-PCR

*C. perfringens* total RNA was isolated as described previously [18] and 2 µg of RNA converted to cDNA using AMV reverse transcriptase (Promega) in accordance with the manufacturer's instructions. The reaction products were diluted fivefold before use in QRT-PCR experiments, as described previously [32]. The QRT-PCR reactions were carried out on an ABI PRISM 7700 sequence detector, in a final volume of 25 µl with SYBR Green PCR master mix (Applied Biosystems), 2 µl of diluted RT reaction as the template and 120 nM of each primer (Table S2). To determine gene copy number, 0.2 ng of genomic DNA was used as the template. Total RNA or genomic DNA of each strain was isolated from three biological replicates, and each sample was assayed in triplicate. The values obtained were normalised to that of the *rpoA* gene for each strain, and the results expressed as a proportion of the wild-type.

## Results

### Identification of functional residues in VirS

**Figure 1:**
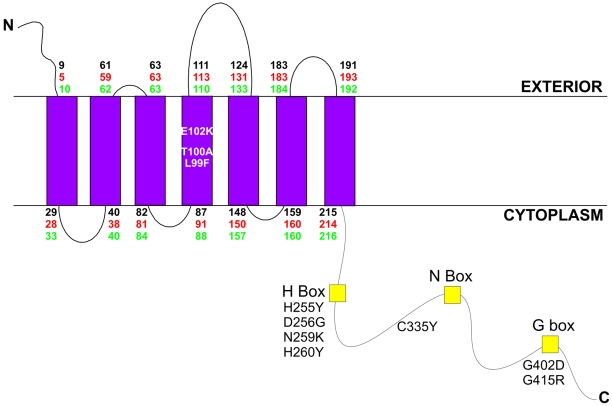
Model of the VirS sensor histidine kinase. The predicted topology of VirS consists of an extracellular *N-*terminus, seven transmembrane domains (blue rectangles) and a cytoplasmic *C*-terminal region. The amino acids encompassing the transmembrane domains as predicted by the TMHMM, Phobius and Sosui algorithms are shown beside the predicted loop regions and are written in black, red and green, respectively. The consensus [39] H box (Fhxxh(S/T/A)H(D/E)h(R/K)TPLxxh), N box ((D/N)xxxhxxhhxNLhxNAh.(F/H/Y)(S/T)) and G box (GGxGLGLxhhxxhhxxxxGxhxhxxxxxxGxxFxhxh) are represented by the yellow squares, while the positions of the random mutations are as indicated.

VirS contains several residues and motifs that are conserved in *C*-terminal domains of sensor histidine kinases, including the putative site of autophosphorylation, H255, and the G (GxGL) motif (Fig. 1). In previous work, six or seven transmembrane regions were predicted in the *N-*terminal region of VirS. In this study, a new model of VirS was obtained using three independent transmembrane prediction algorithms: TMHMM (http://www.cbs.dtu.dk/services/TMHMM/), Phobius (http://phobius.cbr.su.se/) and Sosui (http://bp.nuap.nagoya-u.ac.jp/sosui/). These algorithms showed a consensus topology, where the extracellular *N-*terminus was followed by seven transmembrane domains, and the catalytic *C*-terminal region was cytoplasmically located (Fig. 1). Attempts at verifying this topology using PhoA/LacZ fusions were unsuccessful (data not shown).

With this model in mind, random mutagenesis was used to identify VirS residues of functional importance, particularly within the *N*-terminal domain. Mutagenesis of the *virS^+^* shuttle plasmid pJIR884 [7] led to the isolation of 134 non-hemolytic mutants, each containing plasmids that were indistinguishable from pJIR884 by restriction analysis. Eighteen of these plasmids were found to contain a single point mutation in the *virS* structural gene, 10 of which were unique (Fig. 1) and therefore were subjected to quantitative analysis. None of these mutants conferred any detectable perfringolysin O activity (Table 2), indicating that the mutations had eliminated all measurable VirS function. RT-PCR was used to confirm that the mutated *virS* genes were still transcribed in *C. perfringens* (data not shown).

Three of the VirS substitutions, L99F, T100A and E102K, were located in the *N*-terminal region, within a putative transmembrane domain (TMD 4). The other changes were all located within the cytoplasmic *C*-terminal domain. Four substitutions, H255Y, D256G, N259K and H260Y, were located either in or near H255, the putative site of autophosphorylation. One alteration, C335Y, was close to the conserved N box, a region predicted to form part of the catalytic domain that binds ATP. The G402D and G415R substitutions were located in or near the G box, which is also predicted to form part of the catalytic domain [39].

To define the functional region in this *N*-terminal portion of VirS, several leucine residues on either side of L99, and upstream of E102, were targeted for substitution. The sequence of this leucine rich region is _88_IMISLIFWLFMLTVEAL_104_. The leucine residues adjacent to L99, L104 and L96, were altered to phenylalanine, on the basis that the random L99F mutation eliminated VirS function. The L104F substitution had no significant effect on perfringolysin O production (Table 2), suggesting that this residue is not essential for VirS function. By contrast, the L96F mutation had a dramatic effect on VirS function, eliminating VirS activity. Further mutagenesis of L96 to alanine or asparagine, had no significant effect on perfringolysin O activity, suggesting that the size of the side-chain was the critical factor at this position. To further delineate this putative motif, L92 and I88 were also altered to phenylalanine. These changes had little effect on perfringolysin O activity (Table 2), implying that these residues were not of functional significance.

The amino acid requirements at positions 99 and 100 were also determined by site-directed mutagenesis, the requirement for a glutamate residue at position 102 having been determined previously [18]. The substituted amino acids were chosen so that they had an altered size or charge compared to the original residue in that position. Alteration of L99 to either alanine or asparagine abolished VirS function (Table 2), implying that both the length and hydrophobicity of the side-chain at this position was important. At position 100 a hydroxyl group was required since T100V was inactive, T100C was partially functional and T100S (a conservative substitution) had almost wild-type activity. Based on these data we have designated this region of VirS as the L[T/S]×E motif, and postulate that due to its location it is either required for the conformational change that induces autophosphorylation, or for the structural integrity of VirS.

### Residues in the *C*-terminal domain of VirS are essential for autophosphorylation

**Figure 2:**
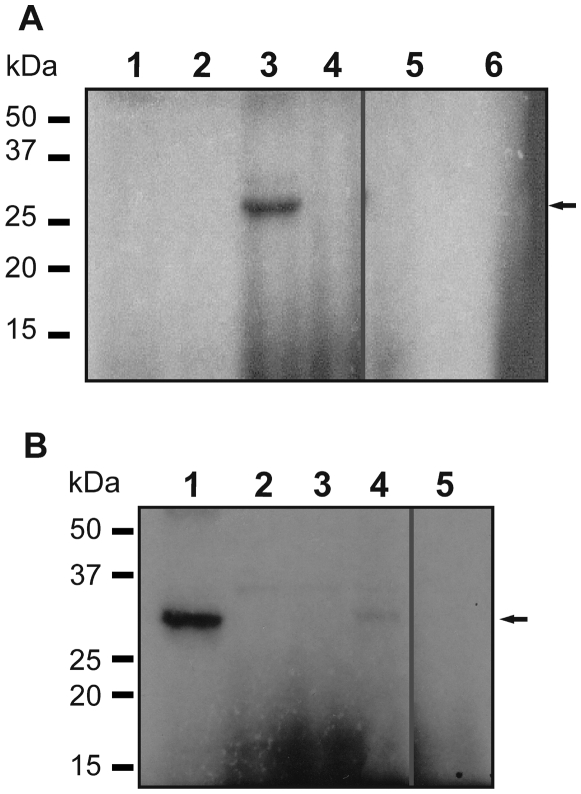
Autophosphorylation of VirSc and its derivatives. (A) In the absence of purified VirSc, a labelled protein band was not observed in the presence of [γ-^32^P]ATP (lane 1) or [α-^32^P]ATP (lane 2). A labelled band of ∼27 kDa was observed when purified VirSc (20 µM) was incubated in phosphorylation buffer in the presence of [γ-^32^P]ATP (lane 3). To show specificity of autophosphorylation, VirSc was incubated with [α-^32^P]ATP (lane 4), or with [γ-^32^P]ATP in the presence of 50 mM EDTA (lane 5) or 2.5 mM unlabelled ATP (lane 6). (B) Autophosphorylation of substituted derivatives of VirSc. VirSc was able to autophosphorylate (lane 1), while VirSc_H255I_ (lane 2), VirSc_C335Y_ (lane 3) and VirSc_G402D_ (lane 5) could not undergo autophosphorylation. VirSc_G400A_ (lane 4) showed severely reduced autophosphorylation. The band corresponding to phosphorylated VirSc (∼27 kDa) is indicated by the arrow. Molecular size markers (kDa) are as indicated.

The next step in our systematic analysis of the VirSR phosphorelay was to determine whether VirS was able to undergo autophosphorylation. The VirS protein is postulated to contain multiple transmembrane domains in the *N*-terminal region, with the catalytic motifs being localised in the cytoplasmic *C*-terminal domain [7], [18]. Since attempts to purify full-length VirS were unsuccessful, a *virS* segment encoding the *C*-terminal domain (amino acids 215 to 440) was cloned into the pET-22b(+) expression vector, and a 27.7 kDa 6×-His tagged protein (VirSc) overexpressed. After purification on a Talon column, VirSc was used in autophosphorylation experiments using [γ−^32^P]ATP or [α-^32^P]ATP. A single band corresponding to the correct size of VirSc (27.7 kDa) was observed in the [γ−^32^P]ATP reaction (Fig. 2A, lane 3), indicating that VirSc was able to undergo autophosphorylation. Moreover, labelling of the protein occurred through the specific transfer of the γ-phosphoryl group from ATP to VirSc, since the protein could be labelled with [γ−^32^P]ATP, but not with [α-^32P^]ATP (Fig. 2A, lane 4), indicating that labelling occurred through a specific phosphotransfer process. The autophosphorylation reaction was found to require divalent metal ions, since pre-incubation of VirSc with 50 mM EDTA inhibited autophosphorylation (Fig. 2A, lane 5). The addition of a 10-fold molar excess of unlabelled ATP competitor also abrogated autophosphorylation (Fig. 2A, lane 6).

In a previous study, mutation of the conserved histidine residue (H255I), or the first glycine residue in the conserved G box (G400A), was shown to eliminate VirS function [18]. Similarly, the C335Y and G402D substitutions of VirS, which were identified through random mutagenesis in this study, also eliminated VirS function (Table 2). To examine what effects these changes had on autophosphorylation *in vitro*, VirSc proteins containing these modifications were purified and tested for their ability to autophosphorylate. The results showed that a phosphorylated protein band was not observed with VirSc_H255I_ (Fig. 2B, lane 2), indicating that this derivative was no longer able to carry out autophosphorylation and that this histidine residue was involved in this reaction. Analysis of the VirSc_C335Y_ derivative showed that this mutation also eliminated the ability of the protein to autophosphorylate (Fig. 2B, lane 3). Therefore, although C335 is located near to, but is not part of the actual N box, it still plays an important structural or functional role in the VirS autophosphorylation process. Significantly reduced VirS-specific autophosphorylation was detected with VirSc_G400A_ (Fig. 2B, lane 4), implying that although the first glycine residue in the G box is important for the autophosphorylation of VirS, very low level phosphorylation is possible when it is altered. However, alteration of the second glycine residue in this motif (G402D) completely eliminated autophosphorylation (Fig. 2B, lane 5). Taken together, these results provided experimental evidence that the motifs and residues predicted to be essential for ATP binding and kinase activity are required for autophosphorylation.

### Phosphotransfer from VirSc to VirR *in vitro*

**Figure 3:**
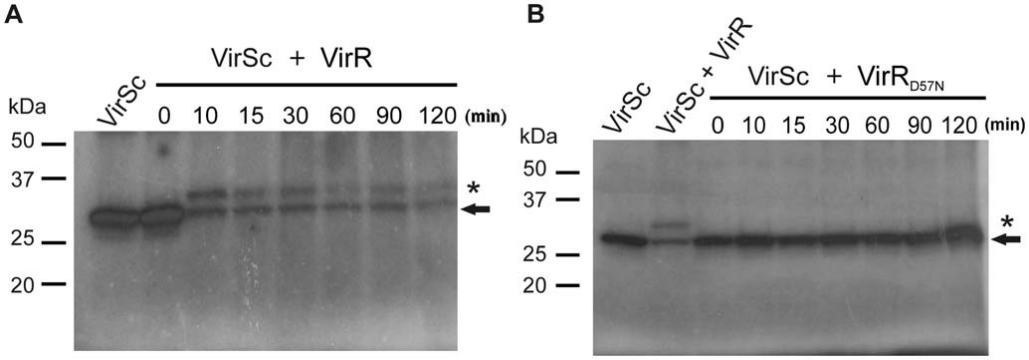
Phosphotransfer from VirSc to VirR. VirSc (10 µM) was incubated in phosphotransfer buffer in the presence of [γ-^32^P]ATP at room temperature before purified (A) VirR or (B) VirR_D57N_ was added. Reactions were incubated at room temperature for the time indicated above each well. The first lane of each gel contains autophosphorylated VirSc, while subsequent lanes show the reactions with both phosphorylated VirSc and VirR or VirR_D57N_. The band corresponding to phosphorylated VirSc (∼27 kDa) or VirR (∼32 kDa) is indicated by the arrow or an asterisk, respectively. Molecular size markers (kDa) are as indicated.

Using phosphorylated VirSc (VirSc-P) as the phosphodonor, we then tested the ability of purified VirR to act as a phosphoacceptor. The appearance of a labelled band corresponding to VirR was observed (Fig. 3A). This result provided evidence for the transfer of the phosphoryl group from VirSc-P to VirR, indicating that VirR was able to interact with the truncated VirS protein. A phosphorylated response regulator band was not observed when purified VirR containing a substitution at the putative D57 phosphoacceptor site (VirR_D57N_) was tested under the same conditions (Fig. 3B). Furthermore, the intensity of this VirSc-P band did not appear to diminish over time. It was concluded that the VirR_D57N_ was not able to be phosphorylated and that D57 was most likely the site of phosphorylation in VirR.

### VirR proteins with mutations in conserved residues are still functional *in vivo*

To determine whether the VirR_D57N_ protein was functional *in vivo*, the *virR_D57N_* gene was subcloned into the *E. coli-C. perfringens* shuttle vector, pJIR750 [40], and the resultant plasmid, pJIR1882, was introduced into *C. perfringens virR* mutant TS133 [8] (Table 1). Since complementation of the *virR* mutation in TS133 with a plasmid carrying a wild-type *virR* gene results in the restoration of perfringolysin O activity [8], perfringolysin O production was used to determine the effect of the mutations. Unexpectedly, when the shuttle plasmid carrying the *virR_D57N_* gene was introduced into TS133, the resultant strain carrying the mutated gene produced less perfringolysin O than the wild-type, but still produced very significant levels of the toxin (Table 3).

**Table 3:** Effect of *virR* genes on perfringolysin O production.

Strain	Characteristics	PFO Titre* (log_2_)
JIR325	Wild-type	7.8±0.4
TS133	*virR* mutant	<1.0
JIR4485	TS133(pJIR750)	<1.0
JIR4508	TS133(pJIR1897), *virR^+^*	8.0±0.5
JIR4487	TS133(pJIR1882), *virR_D57N_* ^+^	6.6±0.4^a^
JIR4488	TS133(pJIR1884), *virR_K105E_* ^+^	7.3±0.3^b^
JIR4618	TS133(pJIR2162), *virR_E8N,D9N,D57N_* ^+^ *(_NNN_ )*	7.9±0.3
JIR12142	TS133(pJIR884), *virS* ^+^	<1.0
JIR12146	TS133 *plc* :: Targetron(pJIR884), *plc virS* ^+^	<1.0
JIR12157	TS133 *plc* :: TargetronΩ*virR*(pJIR751), *plc virR^+^*	4.0±0.7
JIR12154	TS133 *plc* :: TargetronΩ*virR*(pJIR884), *plc virR^+^ virS^+^*	7.8±0.1
JIR12152	TS133 *plc* :: TargetronΩ*virR_D57N_* (pJIR751), *plc virR_D57N_* ^+^	<1.0
JIR12150	TS133 *plc* :: TargetronΩ*virR_D57N_* (pJIR884), *plc virR_D57N_* ^+^ *virS^+^*	<1.0

* PFO titre±SD: Perfringolysin O activity is shown as the average of duplicate assays carried out on preparations from at least three separate cultures of each strain.

a Student's t-test P<0.01, when compared to JIR4508.

b Student's t-test P<0.05, when compared to JIR4508.

Another derivative was subsequently constructed in which the putative essential lysine residue [7] was substituted (K105E). When this mutated *virR* gene was introduced into TS133, similar results were obtained (Table 3). Finally, in case alternate acidic residues in the phosphoacceptor site were being used as phosphoacceptors, a mutant was constructed in which D57 and the other residues in the acceptor pocket, E8 and D9, were substituted. This *virR_E8N D9N D57N_* mutation, referred to as *virR_NNN_,* was also capable of fully complementing the *virR* mutation in TS133 (Table 3). Confirmation that all the plasmids analysed in this strain had retained the expected mutations and had not undergone further *virR* changes was obtained after plasmid extraction and sequence analysis.

### The gene dosage of *virR* is important for the normal regulation of perfringolysin O production

We postulated that this apparent phosphorylation independence occurred as a result of gene dosage effects. To test this hypothesis, we utilised an α-toxin (*plc*)-targeted Targetron vector [37] as a novel means of introducing *virR* genes back onto the chromosome in single copy. Using this method we constructed separate strains in which the wild-type *virR* gene or the *virR_D57N_* gene was inserted into the *plc* gene. As a result of the genetic organisation of the *virRS* operon, the original insertional inactivation of *virR* in TS133 also eliminated the expression of *virS* [41]. Therefore, to reconstitute the VirSR regulatory system, pJIR884, which carries the wild-type *virS* gene [7], was introduced into each of these strains to provide *virS in trans*. Control strains carried the vector plasmid pJIR751.

**Figure 4:**
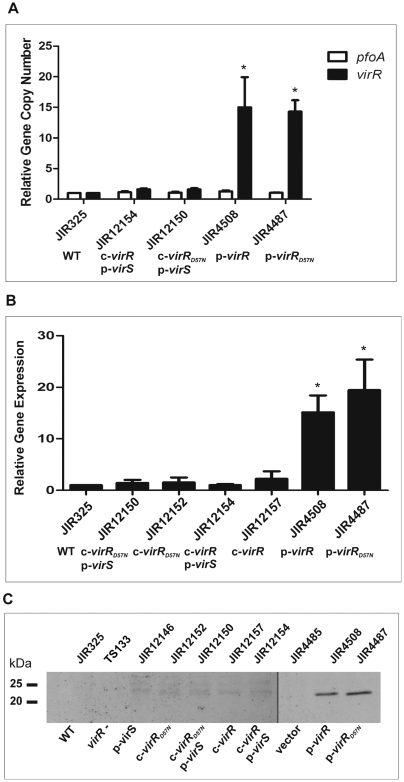
Determination of *virR* copy number, *virR* expression levels and VirR levels in *C. perfringens*. (A) Determination of virR copy number. Genomic DNA from each strain was isolated from three biological replicates and assayed in triplicate by QRT-PCR. The gene copy number of virR (black bars) and pfoA (white bars) in each strain relative to the wild-type (WT) copy number normalised to the rpoA gene is shown. The strains are indicated below each set of bars, and the chromosomal (c) or plasmid (p) location of the virR, virR_D57N_ and/or virS genes is shown beneath each strain. (B) Determination of virR expression using QRT-PCR. Total RNA from each strain was isolated from three biological replicates and assayed in triplicate. The expression levels of virR (black bars) in each strain relative to WT levels normalised to the rpoA gene (relative gene expression) are shown. The strains are indicated as for (A). In (A) and (B), the error bars represent the standard error of the mean, and the asterisks (*) represent a P-value of<0.05 calculated by two-tailed Student's t-test. (C) Immunoblot analysis of wild-type and TS133 derivatives. Western blots were carried out on whole cell extracts of the strains indicated above each lane. A brief description of each strain is given below the blot. The blots were probed with polyclonal rabbit VirR antiserum, followed by chemiluminescent detection. Molecular size markers (kDa) are as indicated.

QRT-PCR using genomic DNA showed that as in the wild-type strain there was one copy of *virR* or *virR_D57N_* in the Targetron-derived chromosomal *plc* insertion derivatives although there were an average of 15 copies of *virR* and 18 copies of *virR_D57N_* in the TS133 strains carrying these genes on plasmids. As an internal control, the copy number of the *pfoA* gene was determined to be one in all of these strains, as expected (Fig. 4A).

Perfringolysin O assays were carried out to determine the phenotypic effect of these different *virR* gene doses. Expression of both the *virR* and *virR_D57N_* genes on multicopy plasmids in TS133 (strains JIR4508 and JIR4487, respectively) resulted in wild-type levels of perfringolysin O activity (Table 3), despite the absence of *virS* in these strains. By use of QRT-PCR analysis, the expression levels of the *virR* genes in these strains were shown to be significantly higher than in the wild-type strain (Fig. 4B). In addition, Western blotting using VirR-specific antiserum showed that there were higher levels of VirR protein in these cells (Fig. 4C). These results indicated that when VirR or VirR_D57N_ was present in the cell at higher concentrations, the response regulator no longer needed to be activated by phosphorylation to be functional. This observation is clearly illustrated by the results with VirR_D57N_, which was not able to be phosphorylated *in vitro*, but was still able to stimulate high levels of perfringolysin O production when encoded on a multicopy plasmid. By contrast, when the *virR_D57N_* gene was introduced onto the chromosome in single copy, as confirmed by genomic QRT-PCR analysis, the resultant strain was not able to produce perfringolysin O (Table 3). The expression levels of the single copy genes, including the wild-type strain, were significantly lower than the genes carried on the multicopy plasmids (Fig. 4B), and the respective protein concentrations were so low that they were not detected by the VirR-specific antisera in Western blots (Fig. 4C). These results suggest that the *virR* gene dosage is an important factor in the regulation of perfringolysin O production.

### Analysis of chromosomal mutants reveals that phosphorylation of VirR is essential for optimal perfringolysin O production

The chromosomal derivatives were also used to assess the effect of the presence of *virS*, that is, the effect of phosphotransfer, on perfringolysin O production (Table 3). The results showed that despite the presence of a plasmid carrying the wild-type *virS* gene, no perfringolysin O activity could be detected in the *virR* mutant TS133, indicating that, as expected, overexpression of *virS* alone could not activate perfringolysin O production. By contrast, perfringolysin O activity was restored to wild-type levels when the *virS* plasmid, pJIR884, was introduced into TS133 complemented with the chromosomal *virR* gene. These results provided *in vivo* evidence that VirS, in the presence of VirR, is required to activate the expression of *pfoA* and were consistent with the *in vitro* phosphorylation data. Analysis of the data obtained with the chromosomal *virR_D57N_* showed that no detectable perfringolysin O activity was observed even when *virS* was present. It was concluded that D57 was an essential residue for VirS-dependent VirR function *in vivo*.

QRT-PCR using total RNA was carried out to determine whether these results were due to variation in *virR* or *virR_D57N_* expression levels. In each of the chromosomal complementation derivatives the relevant *virR* gene was expressed at levels similar to the wild-type (Fig. 4B). Therefore, even though the *virR_D57N_* gene was being expressed at levels similar to wild-type *virR*, the resultant protein was not able to activate the production of perfringolysin O. Furthermore, the presence of a functional *virS* gene in multicopy did not increase the expression of the *virR* genes (Fig. 4B), nor the amount of VirR protein (Fig. 4C). These results suggest that the higher perfringolysin O activity in the chromosomal *virR* complementation derivative was a result of VirS modifying the VirR protein, a process that requires the D57 residue of VirR. Taken together the results provide evidence that *in vivo* phosphorylation is crucial for optimal perfringolysin O production.

## Discussion

Although the VirSR two-component regulatory system is well studied [1], [13], [20], very little is known about the actual phosphorelay cascade. It has always been postulated, but never experimentally demonstrated, that activation of the VirSR system begins with the interaction of an unknown signal molecule with VirS and subsequent VirS autophosphorylation. In this study, we have identified an essential *N*-terminal VirS motif, L[T/S]×E, that may be involved in the activation of VirS after detection of the unknown signal molecule. Further studies would be required to test this hypothesis, once the signalling molecule has been identified. Early studies on toxin production [42], [43], [44] have led to the hypothesis that the VirSR system may potentially be activated by a quorum sensing mechanism involving a secreted molecule called substance A [1], [20]. Although, this elusive molecule has yet to be isolated, recent studies have suggested that it is a cyclic derivative of the quorum sensing peptide (TSACLWFT), which is the secreted product of the *agrBD^CP^* genes [45].

Alternatively, the L[T/S]×E region may play an important role in transmembrane helix packing. Residues such as glutamate, serine and threonine have been found to be crucial in the tight helical packing of membrane bound proteins, which in turn contributes to the stability, folding and subsequently, the function of the protein [46]. Therefore, the introduced changes may have caused a disruption in protein structure, leading to either misfolding or instability of VirS. At present, however, reliable VirS antisera is not available to examine whether protein stability is affected by the mutations. Nevertheless, the observation that not all the residues surrounding the L[T/S]×E motif were required for VirS function implies that the residues in this motif are of functional significance. We have also shown that a truncated VirS protein, VirSc, is able to autophosphorylate, in the absence of the *N*-terminal sensory domain. Autophosphorylation requires divalent cations and is ATP-dependent, with the γ-phosphoryl moiety of ATP being transferred to VirSc. Furthermore, we have shown that the conserved *C*-terminal motifs postulated to be required for autophosphorylation are indeed functional.

The conserved histidine residue in sensor kinases is the site of autophosphorylation [19], [47], where it is suggested to function as the nucleophile that attacks the ATP γ-phosphate [48], [49]. In this study, it was shown that a VirSc_H255I_ protein could not be phosphorylated *in vitro*, which was consistent with the *in vivo* results obtained previously [18], where a *virS_H255I_* gene was not able to complement a chromosomal *virS* mutation. Taken together these results provide good evidence that H255 is essential for VirS function. Although H255 has been postulated to be the site of autophosphorylation, the finding that another histidine residue, H260, also affected protein function suggests that either histidine could potentially act as the site of phosphorylation. However, determination of the actual site was beyond the scope of this study. The glycine residues of the G box were also found to be important. In particular, the second glycine, G402, was shown to be essential, since mutation of this residue resulted in elimination of autophosphorylation. A non-functional *virSc_C335Y_* mutant was isolated in the random mutagenesis experiments and the resultant VirSc_C335Y_ protein was not labelled in the presence of [γ-^32^P]ATP, suggesting that C335 is required for autophosphorylation. The C335Y substitution was located about 10 amino acids *N*-terminal to the proposed N box, which in other systems is important in ATP binding and hence, autophosphorylation [50], [51], [52]. Residues located outside of the conserved motifs have been found to be involved in interactions with the bound nucleotide [53]. Therefore, it is possible that although C335 is not part of the N-box, it may associate with the bound ATP molecule. Alternatively, the effect of the cysteine to tyrosine change could be structural. Note that it was not possible to test the effect of the substitutions in the L[T/S]×E motif on autophosphorylation because we were unable to purify full-length VirS and the truncated VirSc protein did not include these residues.

The *C*-terminal region also appears to be capable of protein-protein interactions with the cognate response regulator, VirR, since phosphotransfer from phosphorylated VirSc to VirR was observed. This result represents the first time phosphotransfer has been demonstrated in this system. The response regulator superfamily is defined by conservation of *N*-terminal residues that are involved in phosphorylation [54]. The key aspartate and lysine residues are invariant in these phosphorylation-dependent regulators. The remaining conserved residues, an acidic pair, a hydroxyl side-chain and an aromatic residue are proposed to have functional roles in the coordination of the essential divalent cation and phosphoryl group. Alignment of VirR with other response regulators suggested that the central aspartate was D57 [7]. In this study, the results provide evidence that D57 is highly likely to be the site of phosphorylation, since phosphotransfer was not observed with VirR_D57N_ under *in vitro* phosphotransfer conditions in which the wild-type VirR protein acted as a phosphoacceptor. In addition, D57 was shown to be essential for VirR function *in vivo*; when the *virR_D57N_* gene was present on the chromosome in single copy, perfringolysin O production was not activated. The results also indicated that other residues in the phosphorylation pocket or elsewhere in the protein were not phosphorylated instead of D57. It is possible that the D57N mutation caused the misfolding of the resulting protein and the associated loss of activity, but the observation that the mutated gene, when present in multicopy, was able to complement the *virR* mutation of the strain argues against this possibility. Unexpectedly, when the wild-type gene was re-introduced back onto the chromosome of TS133, the resultant VirR protein could stimulate a low level of perfringolysin O production in the absence of VirS. This result may be due to phosphorylation of the VirR protein by small molecular weight phosphodonors. This phenomenon has been observed in other two-component systems [55], [56] and involves an alternative phosphoryl moiety, such as acetyl phosphate or carbamoyl phosphate, donating its phosphate group to a response regulator. Phosphorylation by this process is not as efficient as that involving the cognate sensor histidine kinase [57], [58], which may explain why perfringolysin O production was only partially restored.

In some response regulators phosphorylation alters the protein conformation so that steric inhibition is relieved, and consequently, dimerization and DNA binding can occur [59], [60], [61], [62]. In others, phosphorylation is required for interaction with RNA polymerase [63], [64], [65], [66]. We do not know the precise role of phosphorylation in the VirR-dependent expression of the *pfoA* gene, but previous work has shown that phosphorylation is not essential for DNA binding [22], [33]. We postulate that phosphorylation is required to optimize protein-protein interactions between VirR monomers, VirR and RNA polymerase, or both reactions, since binding to DNA alone is not sufficient to activate perfringolysin O production [22].

In conclusion, based on the data presented in this paper, we propose that the regulation of perfringolysin O production in *C. perfringens* is dependent on three factors. The first is the autophosphorylation of VirS following the detection of an unknown signal. This process may involve the L[T/S]×E motif. The second is a requirement for VirS-dependent VirR phosphorylation, a process that involves conserved motifs in both proteins. The third is the *virR* gene dosage in the cell, since overexpression of wild-type and mutated *virR* genes resulted in VirS-independent activation. It appears that the VirSR system is not limited to *C. perfringens*, with genes encoding orthologs of VirR and/or VirS identified in *Clostridium difficile* [32], *Clostridium tetani* [67] and *Clostridium botulinum* [68]. In addition, VirR binding sites have been identified in *C. difficile*, and its VirR ortholog, RgaR, has been shown to recognise and bind specifically to the *C. perfringens* VirR boxes [32]. Therefore, our studies clearly have relevance to the analysis of two component signal transduction system in other pathogenic clostridial species.

## Supporting Information

Table S1 (0.07 MB DOC)

Table S2 (0.06 MB DOC)
