# Supplementary material for: Functional Analysis of the VirSR Phosphorelay from Clostridium perfringens
Source: PLoS One. 2009 Jun 9;4(6):e5849. doi: 10.1371/journal.pone.0005849 (PMC2688746; doi:10.1371/journal.pone.0005849)
Supplement: Table S1 — (0.07 MB DOC) [file pone.0005849.s001.doc]

Table S1: Bacterial plasmids used in this study

| Plasmid | Characteristics | Reference |
| --- | --- | --- |
| pRSET-A | *lacZ’*, F1 origin, ColE1 origin, T*erm*-MCS-EK(His)6 ATG-T7 promoter, Ap^R^ | Invitrogen Corporation |
| pET-22b(+) | Expression vector; *C*-terminal 6-His tag; Ap^R^; 5.5kb | Novagen |
| pJIR750 | *E. coli - C. perfringens* shuttle vector, Cm^R^ | [1] |
| pJIR750ai | pJIR750Ωα-toxin Targetron | [2] |
| pJIR751 | *E. coli - C. perfringens* shuttle vector, Em^R^ | [1] |
| pJIR884 | pJIR872Δ*Hpa*I(0.4kb) | [3] |
| pJIR1342 | pRSET AΩ(*Bam*HI*/Eco*RI: JRP372/371 PCR product, 0.711 kb, *virR^+^*) | [4] |
| pJIR1531 | pBluescriptSK+Ω(*Xba*I/*Hin*dIII: pJIR1342, 0.9 kb fragment containing *virR*) | This study |
| pJIR1732 | pBluescriptSK+Ω(*Xba*I/*Hin*dIII: pJIR1531, 0.9 kb fragment containing D57N mutation in *virR*) | This study |
| pJIR1747 | pRSET AΩ(*Xba*I/*Hin*dIII: pJIR1732, 0.9 kb fragment, *virR_D57N_*) | This study |
| pJIR1775 | pJIR1531 containing *virR_K105E_* mutation | This study |
| pJIR1845 | pUC18Ω(*Bam*HI/*Bgl*II: pJIR870, 1.4 kb, contains *virR* promoter and *virR^+^*, *ΔvirS*) | [5] |
| pJIR1859 | pUC18Ω(pJIR1845; JRP372/UP, PCR product 1.0 kb, contains *virR* promoter region and *virR*) | [5] |
| pJIR1877 | pJIR1859, *virR*(*Bam*HI) | [5] |
| pJIR1881 | pJIR1877Ω(*Bam*HI/*Eco*RI: pJIR1732, 0.71kb, *virR_D57N_*) | This study |
| pJIR1882 | pJIR750Ω(*Hin*dIII/*Eco*RI: pJIR1881, 1.0 kb, *virR_D57N_*) | This study |
| pJIR1883 | pJIR1877Ω(*Bam*HI/*Eco*RI: pJIR1775, 0.71kb, *virR_K105E_*) | This study |
| pJIR1884 | pJIR750Ω(*Hin*dIII/*Eco*RI: pJIR1883, 1.0 kb, *virR_K105E_*) | This study |
| pJIR1897 | pJIR750Ω(*Hin*dIII/*Eco*RI: pJIR1877, 1.0 kb, *virR(*BamHI*)*) | This study |
| pJIR2056 | pUC18Ω(*Sal*I/*Xba*I: pJIR884, 4.3 kb fragment, *virS*^+^) | This study |
| pJIR2118 | pJIR1877 containing *virR_E8N,D9N,D57N_* mutation | This study |
| pJIR2162 | pJIR750Ω(*Hin*dIII/*Eco*RI: pJIR2118, 1.0 kb, *virR_E8N,D9N,D57N_*) | This study |
| pJIR2426 | pET-22bΩ(pJIR1877; JRP1248/1249 PCR product 0.71 kb, *virR^+^*) | This study |
| pJIR2699 | pET-22bΩ(*Nde*I/*Xho*I: JRP1133/1873 PCR product, 0.69 kb, *virSc^+^*) | This study |
| pJIR2792 | pET-22bΩ(*Nde*I/*Xho*I: JRP1133/1873 PCR product, 0.69 kb, *virSc_H255I_*) | This study |
| pJIR2825 | pET-22bΩ(*Nde*I/*Xho*I: JRP1133/1873 PCR product, 0.69 kb, *virSc_G402D_*) | This study |
| pJIR3058 | pET-22bΩ(*Nde*I/*Xho*I: JRP1249/1248 PCR product, 0.71 kb, *virR_D57N_*) |  |
| pJIR3243 | pJIR750aiΩ(*Mlu*I: JRP2812/2813, 0.99 kb fragment containing *virR_D57N_*) | This study |
| pJIR3326 | pJIR750aiΩ(*Mlu*I: JRP2812/2813, 0.99 kb fragment containing *virR*) | This study |

REFERENCES

1. Bannam TL, Rood JI (1993) *Clostridium perfringens-Escherichia coli* shuttle vectors that carry single antibiotic resistance determinants. Plasmid 29: 223-235.

2. Chen Y, McClane BA, Fisher DJ, Rood JI, Gupta P (2005) Construction of an alpha toxin gene knockout mutant of *Clostridium perfringens* type A by use of a mobile group II intron. Appl Environ Microbiol 71: 7542-7547.

3. Lyristis M, Bryant AE, Sloan J, Awad MM, Nisbet IT, et al. (1994) Identification and molecular analysis of a locus that regulates extracellular toxin production in *Clostridium perfringens*. Mol Microbiol 12: 761-777.

4. Cheung JK, Rood JI (2000) The VirR response regulator from *Clostridium perfringens* binds independently to two imperfect direct repeats located upstream of the *pfoA* promoter. J Bacteriol 182: 57-66.

5. McGowan S, Lucet IS, Cheung JK, Awad MM, Whisstock JC, et al. (2002) The FxRxHrS motif: a conserved region essential for DNA binding of the VirR response regualtor from *Clostridium perfringens*. J Mol Biol 322: 997-1011.
