# Supplementary material for: Functional Analysis of the VirSR Phosphorelay from Clostridium perfringens
Source: PLoS One. 2009 Jun 9;4(6):e5849. doi: 10.1371/journal.pone.0005849 (PMC2688746; doi:10.1371/journal.pone.0005849)
Supplement: Table S2 — (0.06 MB DOC) [file pone.0005849.s002.doc]

**Table S2. Oligonucleotide Primers**

| Primer | Sequence (5’→ 3’) | Location/Reference |
| --- | --- | --- |
| **PCR** |  |  |
| UP | GTAAAACGACGGCCAGT | Universal Primer |
| JRP371 | AATAAGGATCCATGTTTAGTATTGCCTTATGTG | Introduces *Bam*HI site at 5’ end *virR* [1] |
| JRP372 | AATAAGAATTCTCATTAACATATTAAATCCCC | Introduces *Eco*RI site at 3’ end *virR* [1] |
| JRP1133 | TTTTCTCGAGGGCTTCTTTTTCTTGATTTA | Introduces *Xho*I site at 3’ end of *virS* |
| JRP1873 | ACCCCCACATATGAAAATAATAAAAAATAAC | Introduces *Nde*I site within *virS* |
| JRP1248 | CCGCTCGAGACATATTAAATCCCCTAAA | Introduces *Xho*I site at 3’ end of *virR* |
| JRP1249 | GGGAATTCCATATGTTTAGTATTGCTT | Introduces *Nde*I site at 5’ end of *virR* |
| JRP2812 | ACGCGTGGGATATGTGAAAAATGGTG | Introduces *Mlu*I site upstream of *virR* promoter region |
| JRP2813 | ACGCGTTCATTAACATATTAAATCCCC | Introduces *Mlu*I site at 3’ end of *virR* |
| JRP2873 | GTGAGGTTATGTTAATTATATGGTATAATTTCAATGC | Upstream of α-toxin Targetron insertion site [2] |
| JRP2874 | AGTTACAATCATAGCATGAGTTCCTGTTCC | Downstream of α-toxin Targetron insertion site [2] |
| **QRT-PCR** |  |  |
| JRP2479 | CCATCTGTTTTTATATCTGCTCCAGTA | Within *rpoA* |
| JRP2480 | GGAAGGTGAAGGACCAAAAACTATT | Within *rpoA* |
| JRP3566 | TTTATAACTGCTTTATGGGATTATATTCAAA | Within *virR* |
| JRP3567 | CCTGCTCTTGTAGCTCCTTAAATT | Within *virR* |
| JRP3385 | GGAGCAATTGATGAGTTAGTGTCTAAGT | Within *pfoA* |
| JRP3386 | TTCTGAATATTGAGTTCTTGCTGGTAA | Within *pfoA* |
| **Site Directed Mutagenesis** | |  |
| JRP912 | GTCGACTCTATAGGATCCCCG | *Xba*I selection primer [3] |
| JRP69 | GTCGACATCGAGGGGGGGG | *Xho*I selection Primer |
| JRP383 | GTGCCACCTGAAGTCTAAGAAACC | *Aat*II selection primer [4] |
| JRP623 | ATGCTTTTTTTAAATATTCAAATGGGTG | *virR* mutagenesis (D57N) |
| #7962 | ATTTAATAGAGCCAGTT | *virR* mutagenesis (K105E) |
| JRP1119 | CTTATGTAATAATAATTCCTTGCAAAGAGAAGAAT | *virR* mutagenesis (E8N,D9N) |

**REFERENCES**

1. Cheung JK, Rood JI (2000) The VirR response regulator from *Clostridium perfringens* binds independently to two imperfect direct repeats located upstream of the *pfoA* promoter. J Bacteriol 182: 57-66.

2. Chen Y, McClane BA, Fisher DJ, Rood JI, Gupta P (2005) Construction of an alpha toxin gene knockout mutant of *Clostridium perfringens* type A by use of a mobile group II intron. Appl Environ Microbiol 71: 7542-7547.

3. Cheung JK, Rood JI (2000) Glutamate residues in the putative transmembrane region are required for the function of the VirS sensor histidine kinase from *Clostridium perfringens*. Microbiol-SGM 146: 517-525.

4. Kennan RM, McMurray LM, Levy SB, Rood JI (1997) Glutamate residues located within putative transmembrane helices are essential for TetA(P)-mediated tetracycline efflux. J Bacteriol 179: 7011-7015.
